# Beyond API-CAT: the remaining challenges of extended anticoagulation for cancer-associated thrombosis

**DOI:** 10.1093/ehjcvp/pvag054

**Published:** 2026-07-22

**Authors:** Marcello Di Nisio, Tzu-Fei Wang

**Affiliations:** Department of Medicine and Ageing Sciences, University G. D’Annunzio Chieti-Pescara, via dei Vestini, snc, Chieti 66100, Italy; Department of Medicine, University of Ottawa at the Ottawa Hospital and Ottawa Hospital Research Institute, Ottawa, ON, Canada

## Abstract

**Graphical Abstract:**
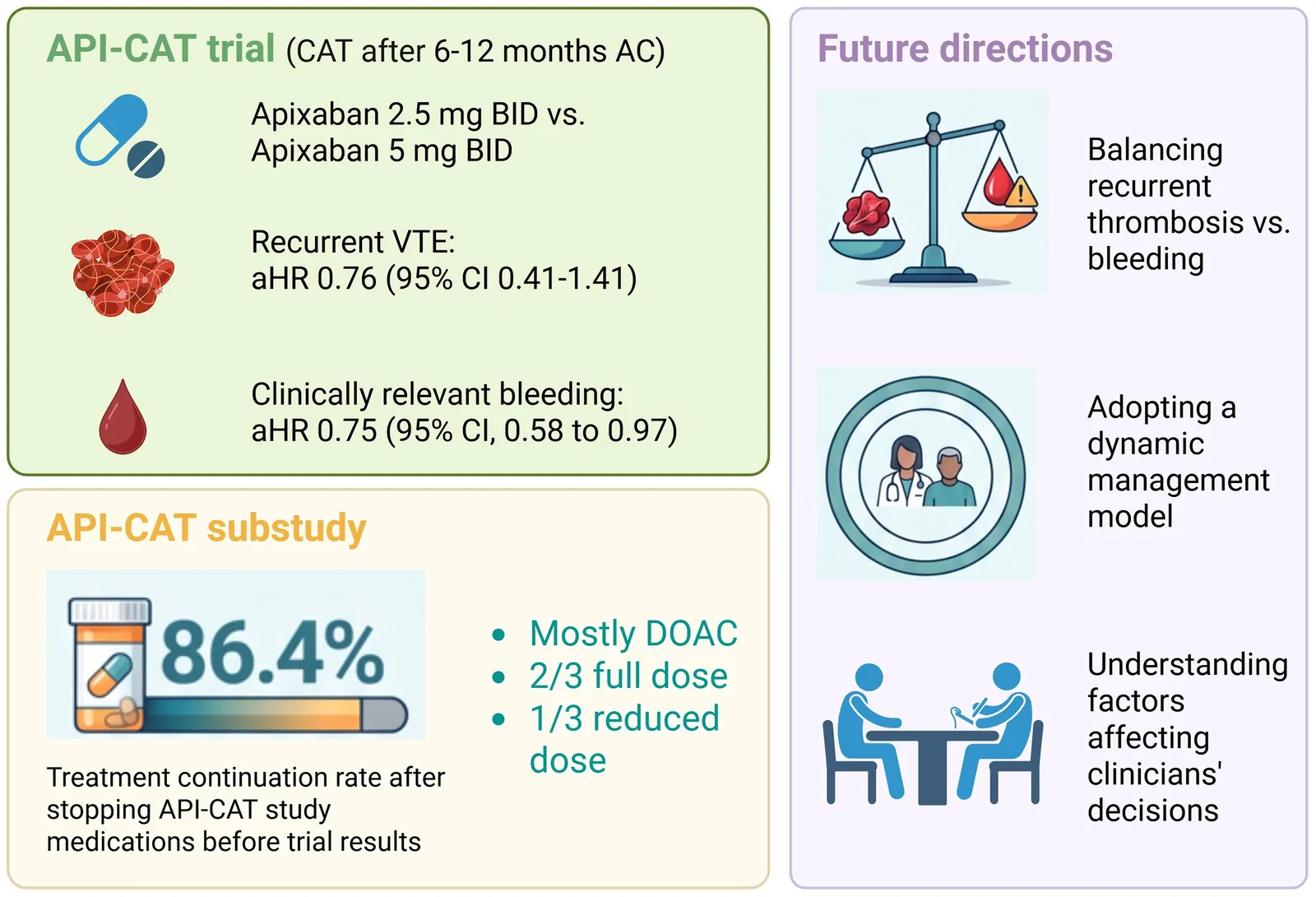

## Introduction

The management of cancer-associated thrombosis (CAT) has changed profoundly over the past decade. Randomized trials have established direct oral anticoagulants (DOACs) as effective alternatives to low-molecular-weight heparin, while improving cancer survival has transformed venous thromboembolism (VTE) from an acute complication into a disease requiring chronic management for many patients with cancer. Current international guidelines recommend continuing anticoagulation beyond the initial 3–6 months in patients with active cancer, provided the bleeding risk remains acceptable.^1,2^ However, these recommendations are largely based on expert opinion and observational evidence rather than randomized data addressing the optimal duration of therapy.

The recently published API-CAT trial represents an important milestone by demonstrating that reduced-dose apixaban (2.5 mg twice daily) is non-inferior to full-dose apixaban (5 mg twice daily) for preventing recurrent VTE beyond 6 months while reducing clinically relevant bleeding in patients with CAT.^3^ These findings will likely establish reduced-dose apixaban as the preferred strategy for extended treatment in many patients with CAT.

In the accompanying issue of the *Journal*, Mahé and colleagues provide a unique snapshot of clinical practice among patients enrolled in the API-CAT trial after completion of the blinded intervention but before trial unblinding and publication of the results.^4^ By prospectively recording investigators’ anticoagulation decisions during this time, they captured expert decision-making in a genuine state of clinical equipoise. Their observations deserve attention not only because they describe practice before API-CAT results changed the evidence base, but because they expose the broader uncertainties that continue to surround extended anticoagulation.

### Extended anticoagulation: progress and remaining uncertainty

The rationale for extended anticoagulation is compelling. Patients with cancer remain at high risk of recurrent VTE long after the initial event. In a recent systematic review and meta-analysis, van Hylckama Vlieg and colleagues reported recurrence rates of 14.6 events per 100 person-years during the first 3 months after anticoagulant discontinuation, with cumulative incidences of 28.3% at 1 year and 35% at 5 years.^5^ These observations strongly support current international guidelines recommending continuation of anticoagulation while cancer remains active.^1,2^

However, prolonged anticoagulation also exposes patients to a persistent risk of bleeding. Even in API-CAT, which enrolled a carefully selected population of patients with CAT who had successfully completed at least 6 months of anticoagulation and were considered suitable for extended therapy, clinically relevant bleeding remained common, occurring in 12.1% and 15.6% of patients receiving reduced-dose and full-dose apixaban over 12 months, respectively.^3^ Although dose reduction significantly improved safety, it did not eliminate bleeding. These findings remind us that extending anticoagulation should not be viewed as a default strategy but as an individualized decision requiring continuous reassessment of the balance between recurrent thrombosis and bleeding.

These observations highlight that decisions regarding extended anticoagulation involve three distinct but closely related clinical questions. Which patients with CAT should continue anticoagulation? For how long should anticoagulation be continued? At what intensity should anticoagulation be administered? API-CAT has convincingly answered the third question. The first two remain largely unresolved.

### What does the study of Mahè and colleagues tell us?

Among patients completing blinded treatment in API-CAT, investigators elected to continue anticoagulation in 86.4%, whereas treatment was discontinued in 13.6%. When anticoagulation was continued, DOACs were overwhelmingly preferred and nearly two-thirds of patients received full therapeutic doses despite the absence of randomized evidence supporting this strategy at the time.

Several observations deserve attention. First, the widespread preference for DOACs reflects the remarkable evolution of CAT management over the past decade, although participation in an apixaban trial undoubtedly contributed to this finding. Second, treatment decisions appeared only weakly associated with measurable patient or cancer characteristics. Exploratory multiple correspondence analysis showed substantial overlap between patients receiving full-dose, reduced-dose or no anticoagulation. Finally, decisions varied according to physician specialty and country, suggesting that clinical practice remained heterogeneous where high-quality evidence was lacking. Collectively, these findings portray a field operating within a genuine therapeutic grey zone immediately before API-CAT results became available.

### Clinical equipoise is not therapeutic uncertainty

One of the most intriguing observations of the study is that treatment choices appeared poorly explained by the measured clinical variables. However, this finding deserves careful interpretation.

Multiple correspondence analysis is an exploratory, descriptive technique that identifies patterns rather than causal determinants of decision-making. The absence of distinct clustering should therefore not be interpreted as evidence that clinicians relied primarily on subjective judgement or local habits. Instead, it may reflect the complexity of modern CAT management. Clinicians simultaneously consider numerous competing factors including tumour type, response to anticancer therapy, previous bleeding, thrombotic burden, frailty, prognosis, renal function, platelet count, and patient preferences, that cannot easily be summarized by a limited number of categorical variables. The observed overlap between treatment groups may therefore reflect individualized clinical decision-making rather than uncertainty driven by non-clinical factors. Indeed, randomized trials estimate average treatment effects, whereas clinicians must make decisions for individual patients.

The unresolved question is not simply treatment duration. API-CAT substantially advances the field by demonstrating that reduced-dose apixaban preserves efficacy while improving safety. Nevertheless, the availability of a safer regimen should not automatically lower the threshold for continuing anticoagulation indefinitely. Even in this highly selected population, clinically relevant bleeding remained frequent despite successful completion of six to twelve months of anticoagulation before enrolment. If bleeding persists in patients carefully selected for a clinical trial, it is likely to remain an even greater concern in routine practice, where frailty, thrombocytopenia, renal impairment and extremes of body weight are more common than in API-CAT. Conversely, discontinuing anticoagulation is not without risks. The high recurrence rates observed after treatment withdrawal in the meta-analysis by van Hylckama Vlieg and colleagues reinforce current recommendations favouring continued anticoagulation in patients with active cancer.^5^ The challenge, therefore, is not deciding whether continuation or discontinuation is universally preferable. Rather, it is identifying the patient in whom the expected reduction in recurrent VTE continues to outweigh the cumulative risks of bleeding, treatment burden and competing mortality.

### Future directions

Current guidelines generally recommend continuing anticoagulation while cancer remains active. Although pragmatic, this binary definition oversimplifies a highly heterogeneous clinical reality. The thrombotic and bleeding risks of a patient with metastatic pancreatic cancer receiving platinum-based chemotherapy differ profoundly from those of a patient with resected breast cancer receiving adjuvant endocrine therapy, despite both fulfilling the definition of ‘active cancer’.

Similarly, treatment decisions should evolve following tumour response, progression, changes in systemic therapy, recurrent bleeding, declining renal function, or limited life expectancy. Rather than viewing extended anticoagulation as a single decision made at 6 months, clinicians should consider it as a process of repeated benefit–risk reassessment throughout the patient’s cancer journey. Such a dynamic approach better reflects the evolving nature of both malignancy and anticoagulant therapy.

Future research should extend beyond comparisons between anticoagulant regimens and focus on individualized treatment duration. Dynamic prediction models incorporating cancer response, biomarkers, competing mortality, bleeding risk, and patient preferences may ultimately prove more valuable than further comparisons between anticoagulant doses alone.

Equally important will be understanding why clinicians make different decisions. The present study offers a rigorous snapshot of expert practice immediately before API-CAT results were available, but the reasons underlying treatment choices, including patient preference, institutional policies, and physician perception of risk, were not prospectively collected. In addition, healthcare system factors, such as reimbursement policies, patient out-of-pocket costs and access to anticoagulants, may also contribute to geographical variation in treatment continuation, and deserve further investigation. Future cost-effectiveness analyses of reduced-dose anticoagulation, incorporating different healthcare reimbursement models, may further inform implementation into routine practice. Combining quantitative analyses with qualitative research could provide valuable insights into clinical decision-making in this therapeutic grey zone. Finally, implementation studies should evaluate how rapidly API-CAT modifies routine practice. The current analysis provides a valuable historical benchmark against which future adoption of reduced-dose anticoagulation can be measured.

## Conclusions

API-CAT represents an important milestone that will likely redefine the standard intensity of extended anticoagulation for CAT. The accompanying investigators’ analysis complements these findings by documenting the considerable clinical heterogeneity that existed before this evidence became available.

However, the study also reminds us that optimizing anticoagulant dose is only one component of extended treatment. The remaining challenges are identifying who should continue anticoagulation, for how long, and how often this decision should be revisited as cancer and patient characteristics evolve.

The future of extended anticoagulation is therefore unlikely to lie solely in safer drugs or lower doses, but in a more dynamic and individualized approach to balancing recurrent thrombosis against bleeding throughout the continuum of cancer care.

## Data Availability

No new data were generated or analysed in support of this research.
